# Change of Gene Structure and Function by Non-Homologous End-Joining, Homologous Recombination, and Transposition of DNA

**DOI:** 10.1371/journal.pgen.1000516

**Published:** 2009-06-12

**Authors:** Wolfgang Goettel, Joachim Messing

**Affiliations:** Waksman Institute of Microbiology, Rutgers University, Piscataway, New Jersey, United States of America; Institut Jean-Pierre Bourgin, INRA de Versailles, France

## Abstract

An important objective in genome research is to relate genome structure to gene function. Sequence comparisons among orthologous and paralogous genes and their allelic variants can reveal sequences of functional significance. Here, we describe a 379-kb region on chromosome 1 of maize that enables us to reconstruct chromosome breakage, transposition, non-homologous end-joining, and homologous recombination events. Such a high-density composition of various mechanisms in a small chromosomal interval exemplifies the evolution of gene regulation and allelic diversity in general. It also illustrates the evolutionary pace of changes in plants, where many of the above mechanisms are of somatic origin. In contrast to animals, somatic alterations can easily be transmitted through meiosis because the germline in plants is contiguous to somatic tissue, permitting the recovery of such chromosomal rearrangements. The analyzed region contains the *P1-wr* allele, a variant of the genetically well-defined *p1* gene, which encodes a Myb-like transcriptional activator in maize. The *P1-wr* allele consists of eleven nearly perfect *P1-wr* 12-kb repeats that are arranged in a tandem head-to-tail array. Although a technical challenge to sequence such a structure by shotgun sequencing, we overcame this problem by subcloning each repeat and ordering them based on nucleotide variations. These polymorphisms were also critical for recombination and expression analysis in presence and absence of the trans-acting epigenetic factor *Ufo1*. Interestingly, chimeras of the *p1* and *p2* genes, *p2/p1* and *p1/p2*, are framing the *P1-wr* cluster. Reconstruction of sequence amplification steps at the *p* locus showed the evolution from a single Myb-homolog to the multi-gene *P1-wr* cluster. It also demonstrates how non-homologous end-joining can create novel gene fusions. Comparisons to orthologous regions in sorghum and rice also indicate a greater instability of the maize genome, probably due to diploidization following allotetraploidization.

## Author Summary

Plant genomes analyzed to date contain 15% or more genes that are arranged in tandem arrays. Tandem duplications are a source for allelic variability since their homologous sequences can serve in recombination events. For example, unequal crossing over between amplified genes can result in contraction and expansion of the array. Tandem gene multiplications are also subject to repeat induced gene silencing (RIGS). Most importantly, gene duplications create the evolutionary potential for genetic novelty (neo- or subfunctionalization). In addition to homologous recombination during meiosis, illegitimate recombination in somatic tissues of plants can create events that potentially can be transmitted through reproductive tissue to further enrich genetic diversity. Here we illustrate the evolution from a single Myb homolog to a multigene cluster that exemplifies the evolution of the maize genome. We used the *p* locus to demonstrate how plant genomes expand by polyploidization, gene duplication, and transposition. We characterized in detail the structural changes at the *p* cluster that resulted from genomic instability. Because structure determines function, we linked genomic rearrangements at the *P1-wr* cluster to functional consequences. At the *P1-wr* locus, structural changes caused regulatory/transcriptional modifications that in turn give rise to phenotypic alterations.

## Introduction

Evolution is based on genome instability. Because genome instability can be detrimental to an individual organism, highly sophisticated mechanisms evolved to maintain genome integrity. Processes to prevent instability, such as DNA damage repair systems, however, are error-prone. Consequently, chromosomal changes are passed onto the next generation and will be tested in evolution on the individual and population level. Species as well as inter-species sequence comparisons reveal the dynamic structure of plant genomes as a consequence of genomic instability. It appears that just a few mechanisms are required to explain genomic instability. Minor or local changes that can cause mutations are associated with inaccurate DNA replication, or DNA repair, or recombination [1]. Replication errors, impairment of base excision and mismatch repair, or error-prone translesion synthesis can lead to base substitutions, micro-insertions and micro-deletions. Micro- and minisatellite instability that results in expansion or contraction of short, repetitive sequences is caused by unequal homologous recombination, replication slippage, or by repair impairment.

More dramatic or global changes in chromosome structure occur when two DNA fragments that were previously unlinked are being joined. Such chromosomal rearrangements include deletions, insertions, duplications, inversions, and translocations, and they can occur by transposition, unequal homologous recombination, or illegitimate recombination [2]. All of these processes involve DNA Double-Strand-Breaks (DSBs) and ligations. Already McClintock demonstrated that chromosomal rearrangements such as translocations, deficiencies, ring chromosomes and end fusions could be consequences of chromosome breaks [3]. DSBs can arise in all tissues at all stages of development and are induced by excision of transposable elements, endonucleases, ionizing irradiation (UV, decay of naturally occurring radioisotopes), reactive oxygen species, and mechanical pulling of dicentric chromosomes. DSBs result in cell-cycle arrest and the recruitment of the DSB-repair machinery. An unrepaired DSB leads ultimately to cell death. Dependent on the phase of the cell cycle, availability of homologous sequences close to the break site, DSBs are repaired by illegitimate recombination (also known as non-homologous endjoining (NHEJ)), homologous recombination (HR), or even a combination of both mechanisms (reviewed in [4]–[6]). During meiosis DSBs are probably exclusively repaired by HR.

NHEJ is a major pathway for DSB repair in somatic tissue. The rejoining of the broken ends via NHEJ is associated with deletions of various sizes, but also insertions of sequences (filler DNA) that are often copied from sites close to the DSB. NHEJ does not require sequence similarities for the incorporation of filler DNA into the break. Taken together, NHEJ does not preserve genetic information and genomic integrity at the break site. Only few cases of filler DNA suggesting a DSB break repair have been reported. HR seems to play a minor role in DSB repair in somatic tissues. Homologous sequences used as template for the repair can be in allelic position (sister chromatid), ectopic, or intrachromosomal. Intrachromosomal homologous recombination is often used to repair a DSB that is caused by the excision of a transposon located between two repeats. The DSB repair results in the deletion of the intervening sequence and one of the repeats [7],[8]. Similarly, this mechanism also generates solo LTRs that are derived from LTR retrotransposons.

Transposable elements contributed tremendously to genome evolution (reviewed in [2],[9],[10]). They have modified single genes by transposing within or adjacent to them. Dependent on the insertion site, mobile elements affect genes in various ways. Despite the fact that transposons were discovered because of their chromosome-breaking features, they are mostly recognized for their mutagenic ability to disrupt gene functions. Transposon insertion as well as excision from a coding region with footprint formation can result in a nonfunctional gene product. They can change transcript processing, for example by providing cryptic donor and acceptor sites. Transposons are a source for *cis*-regulatory elements that can change expression of genes nearby. Insertions in promoter regions, for instance, can add or replace regulatory sequences such that the element gains transcriptional control over the affected gene. While transposons can activate gene expression, they also can cause silencing of adjacent genes. To avoid genomic instability, their hosts epigenetically silence most transposable elements. Silencing by heterochromatin formation is often not limited to the transposon but spreads to neighboring genes as observed in position-effect variegation. Despite transposon silencing mechanisms imposed by the host, transposons play an important role in plant genome expansion. Class I transposons transpose *via* a copy-and-paste mechanism, thereby generating additional elements. But also DNA transposons that employ a cut-and-paste mechanism for transposition increase their copy numbers. They amplify by transposition from a replicated donor site on one of the sister chromatids to a yet unreplicated insertion sequence. Amplification also occurs when a transposon is copied from a template into the empty excision site *via* homology-based gap repair [11].

**Figure 1:**
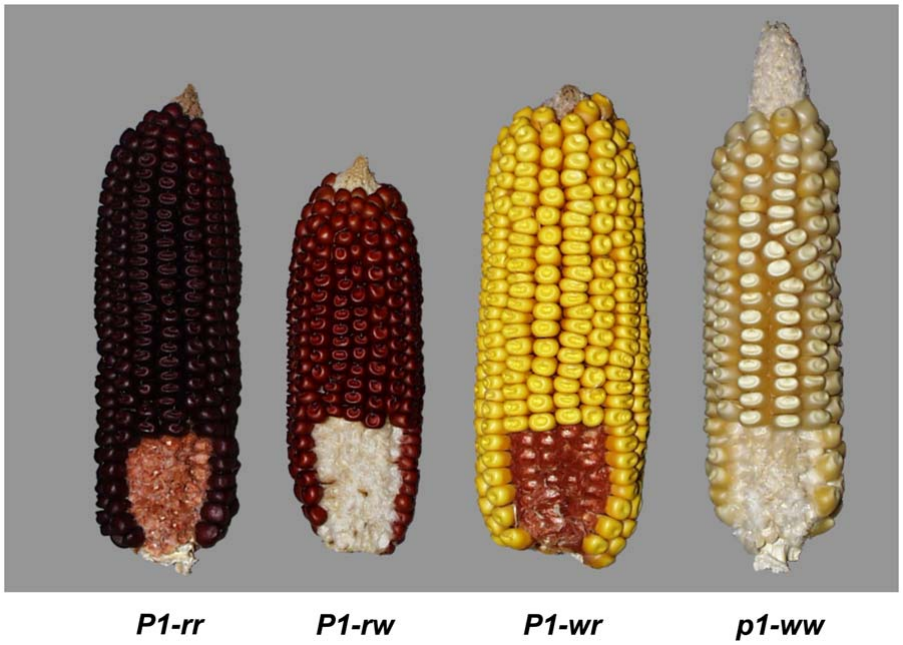
*p1* alleles. *p1* gives rise to phlobaphenes in female floral tissues (pericarp, cob, husks, and silk) and tassel glume margins of the male inflorescence. However, pigmentation is most obvious in pericarp (hence the name of the gene) and in glumes, palea and lemma of the cob. Pericarp or seed coat is the outermost layer of the kernel that is derived from the ovary wall and accordingly is maternal tissue. Glumes, palea and lemma are bracts enclosing the ovary and are also of maternal origin.

To study genomic instability, we favored a gene that is (1) genetically well defined, (2) offers allelic variability, including epigenetically regulated alleles, and (3) has paralogous gene copies. The *p1* gene of maize at the short arm of chromosome 1 (bin 1.03), which encodes an R2R3 Myb-like transcriptional activator meets such properties. *p1* controls the structural genes *c2*, *chi1*, and *a1* of the phlobaphene biosynthesis pathway [12]. Phlobaphenes are reddish flavenoid pigments that are frequently found in male and female maize floral organs. Genes involved in flavonoid pigment biosynthesis are well suited to study numerous aspects of genomic instability because they are dispensable for the organism and generate a visible, quantitative phenotype. Various alleles of *p1* with distinct tissue-specific expression have been collected and investigated. Traditionally, *p1* alleles have been classified phenotypically according to pericarp and glume pigmentation [13]. The *p1* alleles are designated with a two-letter suffix that refers to pericarp and cob coloration, respectively. For example, the *P1*-***rr*** allele displays ***r***ed pericarp and ***r***ed glumes, whereas *P1-* ***wr*** has ***w***hite, or more precisely, colorless pericarp and exhibits ***r***ed glume pigmentation (Figure 1). Although numerous alleles are genetically well described, few of them are sequenced, and often not to completion [12],[14],[15]. The best-studied alleles are *P1-rr* [12] (and its derivatives *P1-vv*, *P1-ovov*, *p1-ww1112*), followed by *P1-wr* [14],[16] and most recently *P1-rw* [15].

The *p1* gene is thought to have arisen by a duplication event from an ancestral *p* gene that is closely related to the recently isolated *p2* gene from a teosinte parviglumis accession [17]. The *p2* gene in maize, which is tightly linked to *P1-rr*, is located proximal of *p1* in the same transcriptional orientation. The teosinte *p2* gene will be referred to as *p2-t* and the maize *p2* gene, which was isolated from a line carrying the *p1* null allele *p1ww1112*, will be designated as *p2-m* throughout the remaining text. In contrast to *p1*, *p2* does not induce visible phlobaphene synthesis in maize tissues [17]. *p2* in teosinte, however, confers pigmentation to tassel glume margins. The gene duplication occurred approximately 2.75 million years ago [17]. Although the name *p2-t* implies the existence of a *p1-t* gene it remains to be seen whether the *p2-t* gene in teosinte is duplicated.

From all alleles of the *p1* gene the *P1-wr* allele is the technically most difficult allele to characterize because of its expanded size and repeat structure. Comparison of different *p1* alleles in maize and its organization in related species of the same family illustrates various molecular mechanisms that have changed entire plant genomes. To distinguish these from lineage-specific events we also compared orthologous regions containing the *p1* locus from rice, sorghum, and the homoeologous region in maize that resulted from an ancient allotetraploidization event.

## Results

### The expanded *P1-wr* locus

**Figure 2:**
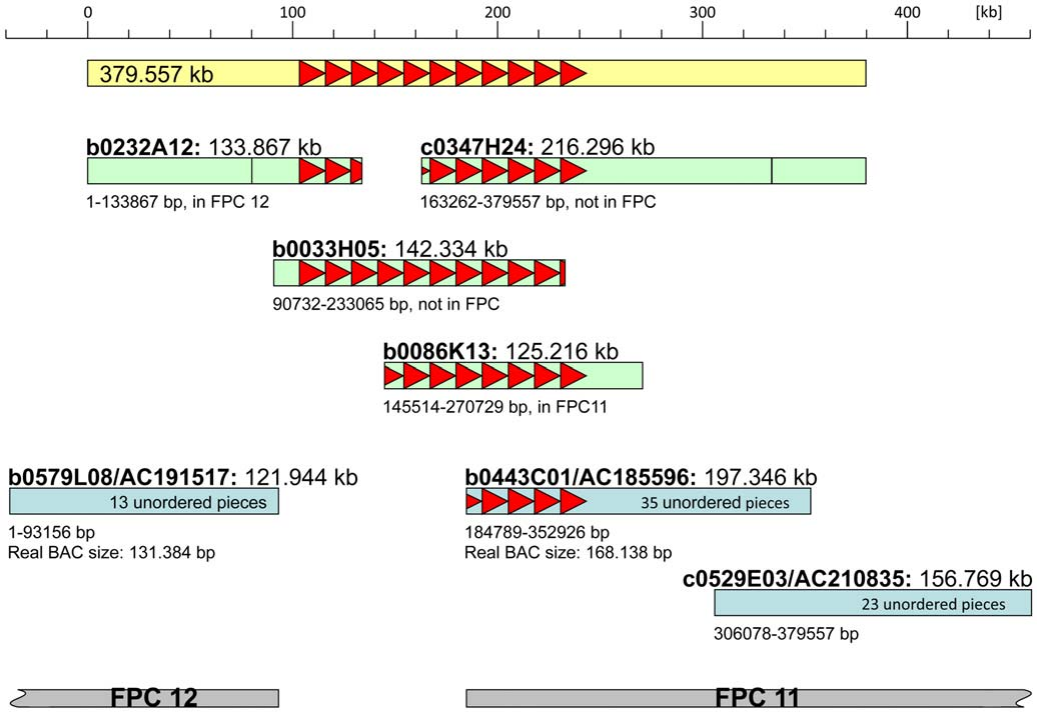
*P1-wr* BACs bridge a FPC and sequence gap. The *p* cluster sequence of 379 kb is represented by a yellow rectangle. Each *P1-wr* repeat is illustrated by a red triangle pointing in the transcriptional orientation of the copy. Individual BACs are displayed as green and blue rectangles, and grey rectangles stand for fingerprinted contigs (FPCs). BACs shown in green were sequenced for this analysis, while BACs in blue were sequenced by a shotgun approach as part of the public maize-sequencing project. Due to the high similarity and large size of each *P1-wr* copy, gaps remain in the *p* cluster for the FPC map and publically available maize sequence as of November 2008. Our sequencing effort bridged the gaps and resolved the structural arrangement of all *P1-wr* repeats. BAC names, their accession numbers and sizes are given on top of each rectangle. Nucleotide positions written underneath the rectangles refer to the *p* cluster sequence that is covered by the BACs. Because BACs shown in blue are not fully assembled, their contig numbers are indicated in the rectangles. Therefore the BAC size corresponds to all added contigs. The calculated BAC size, which is written under the rectangle when available, can be smaller or larger dependent on overlaps or sequence gaps. Information whether BACs shown in green were fingerprinted and assembled in an FPC map is given underneath the rectangles. The vertical lines in two BACs represent a sequence gap in a CACTA and retro element.

**Figure 3:**
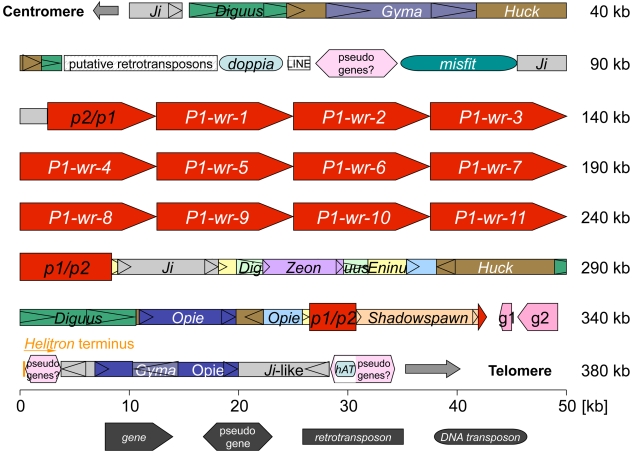
Representation of the *P1-wr* cluster and flanking sequences. *P1-wr* repeats, as well as flanking *p* genes are depicted as red pentagons with the apex pointing in the direction of transcription. Two predicted genes (pink pentagons) that encode a calmodulin binding protein (g1) and an expressed protein (g2) are positioned downstream of *p1/p2*. Regions containing probable pseudogenes are illustrated as pink hexagons. The fragmented genes downstream of the predicted genes are associated with a *Helitron* 3′ terminal sequence. Class I and class II transposable elements (drawn as rectangles and rounded rectangles, respectively) include mostly nested LTR retrotransposons, two CACTA elements (*misfit* and *doppia*), one *hAT* element, one LINE element and several MITEs (not shown). LTRs of retrotransposons are represented as triangles indicating the transcriptional orientation. Notice that the 3′ end of *p1/p2* is separated from the coding region by a large retroelement block. Transposons depicted in white are not well conserved. *P1-wr* repeats are displayed in transcriptional orientation from left to right, while *p2/p1* is proximal and *p1/p2* is distal to the centromere.

Molecular analysis of the multi-copy *P1-wr* allele (Figure 1) required the isolation of three overlapping BAC clones from inbred line B73 (Methods). The large size of this locus is due to nearly perfect tandem genic repeats. The structural analysis of such repetitive genomic sequences requires a different strategy than the standard shotgun sequencing approach. Indeed, the *P1-wr* structure is not available from the current Maize Genome Sequencing Project. The *P1-wr* repeats created a gap in the minimum tiling path that was closed with our contiguous *P1-wr* sequence permitting the merger of finger-printed contigs (FPCs) 11 and 12 (Figure 2) [18]. Sequencing required subcloning of individual repeats using conserved restriction sites and then ordering repeats based on nucleotide polymorphisms [19]. Using such an approach, we identified eleven *P1-wr* copies ranging from 12,602 bp to 13,026 bp in size in a contiguous sequence of 379 kb (Figure 3). The *P1-wr* repeat is defined as the sequence starting from the first nucleotide downstream of exon 3 of the previous copy to the last nucleotide of exon 3. The *P1-wr* repeats, which are highly similar (see below) are named according to their order in the array, starting with *P1-wr-1* for the most 5′ repeat. All numbers here refer to *P1-wr-1*, which was analyzed as a prototype *P1-wr* repeat. *P1-wr-1*, which is 12,648 bp in size, comprises 6,314 bp of transcribed sequence and a 6,334-bp region upstream of the transcription start site. Unless otherwise noted, nucleotide positions given for the *P1-wr-1* sequence refer to the transcription start site.

The *P1-wr* repeats are flanked by two genes that resemble *p2* (Figure 3). Immediately upstream of the first *P1-wr* repeat is a chimeric *p* sequence that consists of a *p2*-like 5′ end, followed by a *P1-wr* 3′ end. Therefore, this sequence will be designated as *p2/p1*. The sequence located downstream of the most distal *P1-wr* repeat resembles *P1-wr* in the regulatory regions and *p2-m* in the majority of the assumed transcribed part. Accordingly, this potential gene will be named *p1/p2*. The 3′ end of *p1/p2* is displaced by numerous retroelement insertions. Besides *p*-related genes, the analyzed sequence includes only two more predicted genes, which are located downstream of *p1/p2*, one potentially encoding a calmodulin binding protein, the other an expressed protein, based on EST data and their syntenic positions in rice and sorghum (see below). In addition, several pseudogenes are present in the *P1-wr* cluster region. The gene encoding an expressed protein is upstream of a *Helitron* terminal sequence, which can form a hairpin structure. The pseudogenes downstream of this *Helitron* terminus are potentially fragmented genes incorporated in the *Helitron* transposon. The cluster contains various transposable elements such as multiple LTR retrotransposons inserted mostly in a nested fashion, two CACTA elements (*misfit* and *doppia*), one *hAT* element, one LINE element and several MITEs. *p2/p1* is proximal, *p1/p2* is distal to the centromere (Figure 3).

### The regulatory and coding regions of the *P1-wr* allele

**Figure 4:**
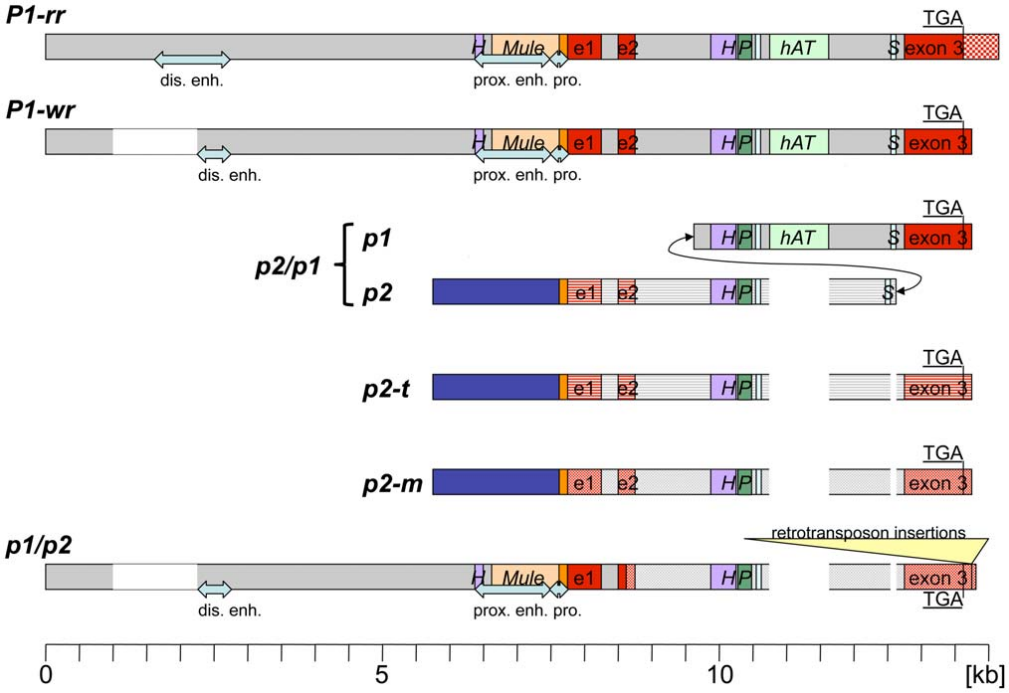
Schematic alignment of *p* genes. While *P1-wr* is the only described *p1* allele with a multi-copy structure, only one copy is shown here. The *P1-wr* 5′ region aligns well with other *p1* alleles. Regulatory elements, *i.e.* distal and proximal enhancer and basal promoter, depicted as blue arrows, were only determined for *P1-rr*. In other *p* genes or alleles, the arrows merely refer to sequence homology to *P1-rr*. Functional homology has not been investigated. A *Heartbreaker* MITE (purple bar) and a *Mu*-like element (tan bar) are part of the proximal enhancer. The *p2* sequences upstream of the transcription start site depicted as blue rectangles are nearly identical. Notice that *p2* shares the initial promoter sequences (orange rectangle) with *p1* alleles. Upstream of *p2*, maize and teosinte differ in their composition of retrotransposons (not shown). The transcribed component of *p1* and *p2* genes (with the exception of the *P1-rr* allele) consists of 3 exons (illustrated in red) and 2 introns. The fourth exon of *P1-rr* is not displayed. *P1-rr* differs from *P1-wr* in the 3′UTR. The *p2* genes from maize (dotted) and parviglumis (horizontal lines) are very similar to *p1* alleles in their transcribed regions. All other sequences shown are hybrids between *p1* and *p2*. The 5′ region of *p2/p1* containing exon 1 and 2 (horizontal red lines) is of *p2* origin while the 3′ end including exon 3 (full red) is derived from *P1-wr*. *p1/p2* switches from a *p1* to a *p2* sequence in exon 2. The 3′ UTR of *p1/p2* was separated by retrotransposon insertions as indicated by two parallel lines. Intron 2 comprises numerous transposable elements of various kinds: a *hAT*-like element (light green) and several MITEs or repeat elements ((*S*) *Stowaway*, (*H*) *Heartbreaker*, (*P*) *Pilgrim*, unnamed MITE).

As shown for *P1-wr* [W23] in transgenic studies, the upstream sequence contains all regulatory elements necessary for expression in pericarp and cob [20]. In *P1-rr*, a 235-bp fragment immediately upstream of the transcription start site has basal promoter functions (Figure 4) [21]. The corresponding fragment in *P1-wr-1* is identical to *P1-rr* excluding a 19-bp and 36-bp insertion in *P1-wr-1*. A 1-kb *Hin*dIII-*Sal*I fragment upstream of the basal promoter was previously identified as a *P1-rr* enhancer. This sequence is well conserved in *P1-wr-1* varying only in four SNPs and six 1-bp indels. Structural analysis of this potential *P1-wr-1* enhancer revealed a complex sequence composition that includes two almost perfect inverted repeats (IR) of 199 and 186 bp separated by 362 bp, which originated from a previously uncharacterized *Mu*-like transposable element that became truncated after insertion in the *p1* gene. Further details about this new element family are provided in the supplemental material (Text S1, Figure S1). This *Mu*-like transposon (position -102 to -1072) occupies the greatest part of sequences defined as the proximal enhancer (position -291 to -1301) and promoter (position -1 to -290) (Figure 4). The element in the proximal enhancer region of *p1* contains a sequence inserted between both TIRs, derived from the first intron of a calcium-dependent protein kinase gene on chromosome 10. This structure and various truncated derivatives are present seven more times in the B73 genome. Compared to the transposon that is closest to the capturing event of the intron, the element in *p1* lacks 226 bp of the 5′ TIR including the TSD and 200 bp from the center of the gene fragment. The TIR deletion break point is adjacent to a 15-bp direct repeat. A *Heartbreaker* MITE can be found 122 bp 5′ of the complex IR structure or 1,195 bp upstream of the transcription start site. Therefore, it appears that multiple double-strand breaks and repairs have to be invoked to compose a regulatory sequence consisting of a truncated *Mu*-like transposon and part of a MITE that are separated by 122 bp. Though nearly identical, *P1-wr-1* differs from *P1-rr* in a region that contains the distal enhancer of *P1-rr* because it shares only a 408-bp sequence corresponding to the 3′ end of the distal enhancer of *P1-rr*. This sequence is located 4,886 bp upstream of the transcription start site (position -4,886 to -5,293). Despite this truncation, *P1-wr* regulatory sequences contain all elements necessary for expression in pericarp tissue [20].

The exon-intron boundaries of *P1-wr* repeats in B73 can easily be defined using the *P1-wr* cDNA from inbred W23, as well as sequence alignments with the *P1-rr* allele. The transcript of *P1-wr-1* is 1,610 nucleotides in size. *P1-wr-1* [B73] is identical to *P1-rr* in the first exon, except for one non-synonymous substitution that converts the fourth amino acid residue alanine in *P1-wr* to threonine in *P1-rr*. This change does not affect the Myb domain of the P1-wr protein, which starts at the 12^th^ residue. The second exon, which is only 130 bp in size, is identical in *P1-wr-1* [B73] and *P1-rr*. *P1-wr-1* [B73] and *P1-rr* do not differ in the coding sequence of exon 3. Besides the substitution of the fourth amino acid residue, the deduced P1-wr-1 [B73] and P1-rr proteins are identical. The proteins, which are 335 amino acids in length, contain a conserved R2R3 Myb domain and a P protein specific activation domain. The Myb domain is located at the N-terminus including residues 12–115. The acidic activation domain is 44 amino acids in size starting with residue 201. *P1-wr* intron sequences are described in the supplemental material (Text S1).

### *P1-wr* repeats are polymorphic

**Figure 5:**
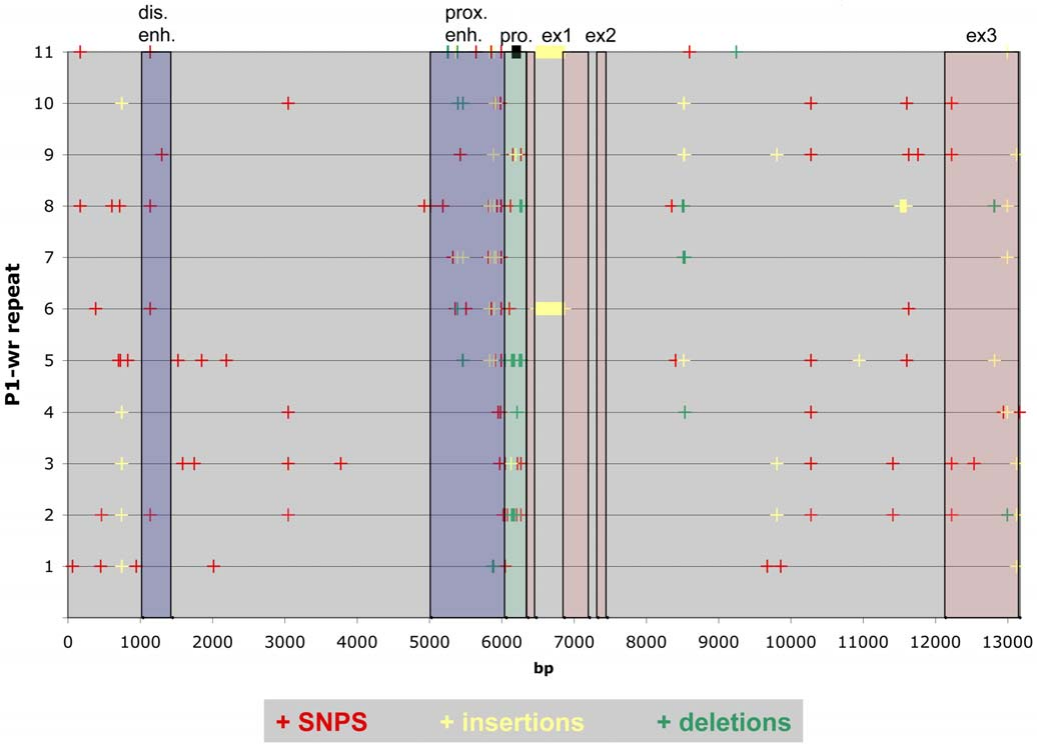
Polymorphisms among *P1-wr* repeats. This plot displays polymorphisms in individual *P1-wr* repeats compared to a *P1-wr* consensus sequence. Polymorphisms are subdivided in separate classes: SNPs are depicted in red, insertions in yellow, and deletions in green. Exons are shaded in red, a putative basal promoter region in green, and potential enhancer sequences in blue. Notice that exon 1 is split by a *hAT*-like transposable element in *P1-wr* repeat 6 and 11.

The alignment of *P1-wr* repeats reveals 103 polymorphic sites, spread over regulatory and transcribed sequences. Compared to the *P1-wr* consensus sequence of 13,172 bp, *P1-wr* repeats differ in 67 SNPs, 20 insertions and 16 deletions (Figure 5). Two indels have features of a remnant *hAT*-like transposable element. Polymorphisms that are shared by at least two *P1-wr* repeats indicate amplification of the *P1-wr* cluster by recombination (unequal crossovers) and/or gene conversion. 13 SNPs, 7 insertions, and 2 deletions are present in more than one *P1-wr* repeat. Still, long stretches of complete identity cannot be detected, demonstrating extensive reshuffling of the *P1-wr* repeats by numerous recombination events. Polymorphic sites are not evenly distributed across the *P1-wr* repeats. Interestingly, the most frequent mutations among the *P1-wr* copies can be found in both TIRs of the *Mu*-like element that contributes to the proximal enhancer and promoter region, although preliminary results indicate no effect of these polymorphisms on transcription rate. The alignment of *P1-wr* sequences exposes in *P1-wr* copies 6 and 11 a large insertion of 382 bp in the 5′ UTR of the first exon, 115 bp after the transcription start site. The insertion sequence exhibits features of an *Ins2* transposable element as described in the supplemental material (Text S1). Because two *P1-wr* repeats contain this insertion, the transposition event probably occurred during *P1-wr* amplification. With exception of the *Ins2* insertion, all repeats are identical in the first and second exon. Four SNPs, three insertions and two deletions are located in the larger exon 3 that can be used to distinguish *P1-wr* transcripts. All SNPs and most indels have no effect on the deduced protein sequence. Only a TGC-insertion in *P1-wr-5* and a TGC-deletion in *P1-wr-8* adds and deletes one alanine from a seven-alanine repeat at the C-terminal end, respectively.

### Transcript analysis

Polymorphisms in *P1-wr* transcribed regions enable us to investigate which repeats are expressed. In addition, differences in coding sequences are most informative regarding gene products. Total RNAs were extracted from pericarp tissue and reversed transcribed. Several primer pairs were used in PCR reactions that amplify polymorphic sequences of all *P1-wr* repeats, *p2/p1*, and *p1/p2*. Genomic DNA served as a control for presence and ratio of individual *P1-wr* repeats. The amplified products were cloned, sequenced, and analyzed. Using primer pair p-ex3-3 that spans four *P1-wr* repeat polymorphisms in exon 3, four out of eleven *P1-wr* repeats and *p1/p2* can be distinguished based on SNPs and indels in the amplified region (Figure 5). By utilizing different primer pairs in the same approach, the transcript analysis revealed that at least eight *P1-wr* repeats and *p1/p2* are expressed (data not shown). Included are *P1-wr* repeats 6 and 11 that contain a *hAT*-like element in the 5′UTR of exon 1. Interestingly, the *hAT* insertion interferes with the correct splicing of intron 1 and prevents synthesis of functional transcription factors. We also investigated expression of *P1-wr* repeats in the presence of *Ufo1*, an epigenetic modifier of *P1-wr*. Consistent with increased pigmentation levels in *p1*-expressing tissues by several fold [22], we found that *p1* transcript levels in an *UfoI* mutant background are augmented (data not shown). However, none of the individual *P1-wr* repeats analyzed here seems to be preferentially activated in the *UfoI* background, indicating a mechanism that affects all *P1-wr* repeats in a similar way.

### The *p2/p1* chimeric gene contains filler DNA

While the SNPs in the *P1-wr* repeats indicate homologous recombination events during meiosis, the chimeric *p2*/*p1* gene upstream of the *P1-wr* repeats (Figure 3) arose by an entirely different mechanism. Until the end of the first repeat, *p2/p1* is most closely related to *p2-t* (84.5% nucleotide identity). Downstream at the 3′ end, *p2/p1* closely resembles *P1-wr* (Figure 4). This gene copy is expressed in silk (data not shown) and encodes a protein identical to P1-wr. Based on the *p2* regulatory sequences of *p2/p1*, *p2/p1* and *p2* are transcribed together in the same tissues at the same time. Although its regulatory sequences have not yet been determined, the initial 92 bp (counting from the transcriptional start site), *p2-m* and *p2-t* are 88% identical to the basal promoter of *p1* alleles. Because the 92-bp fragment is the only sequence common in *p2* and *p1* and silk is the only tissue where both *p1* and *p2* are expressed, it is conceivable that the 92-bp sequence contains a regulatory element necessary for gene expression in silk.

**Figure 6:**
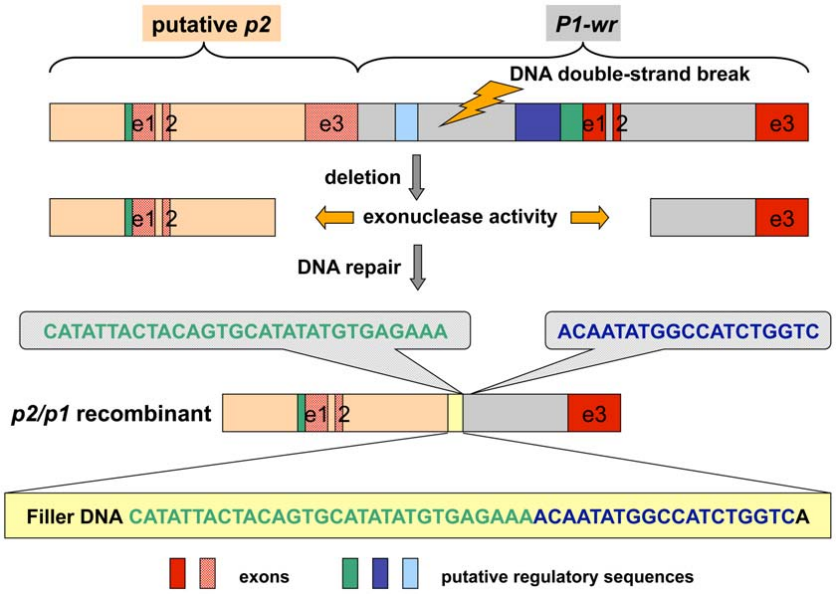
Origin of the chimeric *p2/p1* gene by NHEJ. The recombinant *p2/p1* gene is represented as a rectangle above the filler DNA sequence shown in a yellow box. Exons 1 and 2 from *p2/p1* are similar to *p2*, but the third exon is derived from *P1-wr*. The complex filler DNA (yellow rectangle) originated from two nearby downstream sequences as indicated by two gray balloons. A potential ancestral sequence, consisting of a putative *p2* gene (tan rectangle) and a neighboring *P1-wr* gene (gray rectangle), is shown on top. DNA double-strand break, deletion and repair events resulting in the *p2/p1* recombinant are explained in the main text.

Although in exon 1, intron 1, and exon 2 *p2/p1* resembles *p2-t* more than *P1-wr* or *P1-rr*, the structure of the second intron differs remarkably from previously investigated *p* alleles. Intron 2 with 7,000 bp is significantly longer than in other *p1* and *p2* genes. It contains large direct imperfect repeats, 2,377 bp and 2,701 bp in size, beginning at 1,759 bp and 4,186 bp after the transcription start site, respectively. Both repeats are separated by a 50-bp sequence of known origin (Figure 6). The first 30 bp (CATATTACTACAGTGCATATATGTGAGAAA) are identical to the initial sequence of the second repeat (4,186–4,215 bp after the transcription start site). This sequence is followed by 19 bp (ACAATATGGCCATCTGGTC) that are also derived from the second repeat few nucleotides downstream of the first duplicate (4,278–4,296 bp after transcription start site). The 50^th^ nucleotide A is unaccounted for. The 50-bp sequence can be clearly assigned to the second repeat, due to SNPs between the repeats. This 50-bp sequence is suggestive of filler DNA, which is associated with NHEJ and, therefore, *p2/p1* originated from somatic tissue.

### Chromosomal rearrangements caused by transposition events

**Figure 7:**
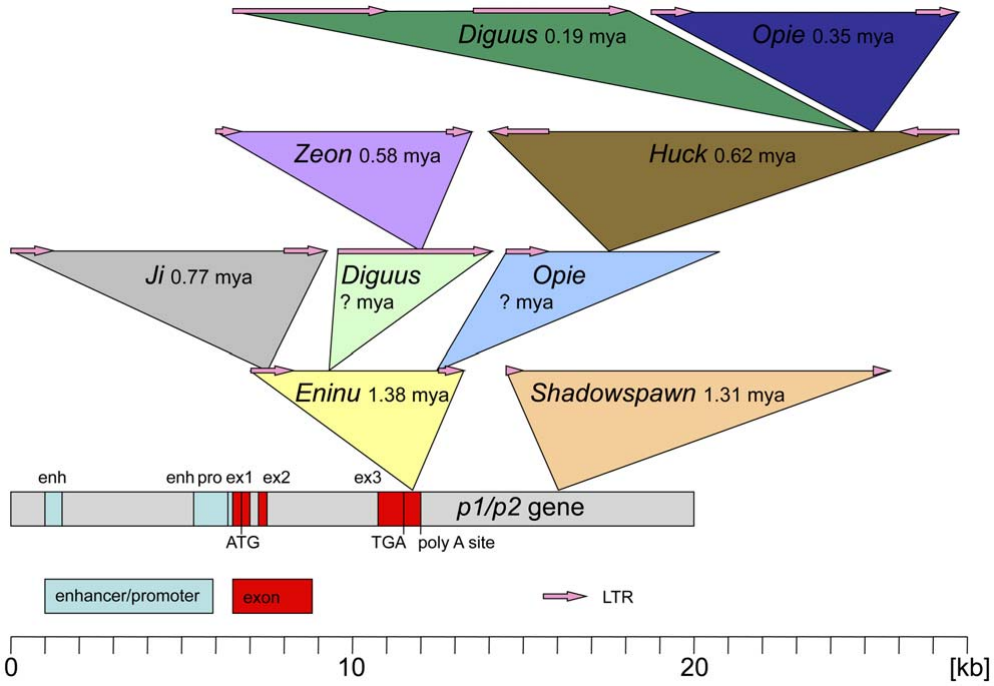
Retrotransposon insertions displaced the *p1/p2* 3′ UTR. The structural organization of the *p1/p2* gene (including exons and putative regulatory sequences) is shown at the bottom of this figure. The first retroelement that inserted approximately 1.38 million years ago (mya) in the 3′UTR of *p1/p2* was *Eninu*. *Shadowspawn* transposed “shortly” after (1.31 mya), in a region 4.1 kb downstream of *Eninu*. *Ji* jumped into *Eninu* 0.77 mya. Based on the nested nature of insertions, an *Opie* element that was truncated later must have inserted after *Eninu* but before *Huck*, which entered *Opie* 0.62 mya. Similarly, *Diguus*, now being a solo LTR, must have inserted into *Eninu* before *Zeon*, which was integrated into *Diguus* 0.58 mya. Finally, *Opie* and *Diguus* jumped into *Huck* 0.35 mya and 0.19 mya, respectively, pushing both ends of *p1/p2* to the total of 68 kb apart. The order of the transposition events can be inferred by the nested nature of insertions, which is consistent with all computed insertion dates.

The chimeric *p1/p2* gene downstream of the most distal *P1-wr* repeat is 98% identical at the nucleotide level to *P1-wr* and *P1-rr* (Figure 4) and is described in more detail in the supplemental material (Text S1). While the upstream chimeric *p2*/*p1* gene is unusual because of the presence of a filler DNA, this chimeric gene has an unusual 3′ end. 247 bp after the stop codon, the homology to *P1-wr* is completely lost. Like in *p2-m* and *P1-rw*, an *Eninu* retrotransposon LTR follows the point of divergence in *p1/p2* (Figure 7). The 5′ end of the *Eninu* LTR is difficult to identify because it is not well conserved relative to other *Eninu* LTRs. The endpoints of *p2* and *P1-rw* transcripts have not been determined yet. The *Eninu* insertion happened upstream of a putative polyadenylation signal sequence AATAAA used for transcript termination. Displacement of this hexamer sequence in *p1/p2* and *p2-m* requires a sequence in the retroelement that substitutes for the original poly-A signal element.

The *p1/p2* sequence continues in the 3′ UTR 68,190 bp further downstream, precisely where the homology to *P1-wr* and *p2-t* stopped before, indicating that a large insertion split off the *p1/p2* 3′ end (Figure 7). This insertion is bordered downstream by an *Eninu* retrotransposon LTR, which lacks approximately 700 bp from the 5′ end. Both *Eninu* LTRs are flanked by identical 5-bp sequences AAGAC, which identifies them as target site duplication sequences (TSD) caused by LTR retrotransposon insertion. This suggests that these LTR pairs belong to the same *Eninu* retroelement (see below). The remaining 61 bp of exon 3 are followed by 4,100 bp 99.2% identical to the initial sequences of a *P1-wr* repeat (beginning with 6,300 bp upstream of the transcription start site). This displaced *P1-wr*-like sequence reveals similar features and structures to *P1-wr*, including the distal enhancer region. The homology is interrupted by the insertion of a single LTR retrotransposon *Shadowspawn*. The homology to *P1-wr* terminates 769 bp after the LTR retrotransposon insertion. Interestingly, the homology to *P1-wr* ends exactly 5 bp before a fragmented *Mu*-like transposable element that has been shown to be part of the proximal enhancer of *p1* alleles (see above). Accordingly, proximal enhancer, promoter, and coding sequences are missing compared to a full length *P1-wr* repeat.

Nine LTR-retrotransposons have been identified in the *P1-wr* cluster, not taking into account retroelements in flanking regions (Figure 7). Six retrotransposons have complete and conserved pairs of LTRs, allowing the use of the total LTR lengths for the dating of their divergence. The initial 700 bp of the 3′ LTR of the *Eninu* element are missing. Thus, only the remaining 635 bp from both LTRs were considered for the calculation of K. One of the *Opie* elements contains only one LTR and almost 5 kb of internal sequence. One of the two *Diguus* retrotransposons consists of only one LTR. The insertion time cannot be determined for the latter incomplete elements. Eight out of nine retroelements are inserted in a nested fashion as described for the *Adh1-F* region previously [23]. Intriguingly, eight out of nine LTR retrotransposons are inserted in the same transcriptional direction suggesting a preferred, rather than random, orientation upon insertion.

### Orthologous genomic regions in maize, sorghum, and rice

The structural organization of the *P1-wr* cluster in maize is rather complex, involving the *p2* gene and multiple copies of the paralogous *p1* gene in a head to tail array. However, single copy *p1* alleles such as *P1-rr* [24] and *P1-rw* [15] have been described previously. To analyze the highly dynamic *p* locus in an evolutionary context, we searched for orthologous gene copies in the close relatives, rice and sorghum, and in the homoeologous chromosome segment of maize. In rice (*Oryza sativa*, japonica cultivar Nipponbare), the sequence most similar to a single maize *P1-wr* gene can be discovered on the short arm of chromosome 3 (position 10,763,678 bp–10,757,863 bp) [25]. Sequence alignments identify a single coding region that is 68.8% identical at the nucleotide level with *p1*. Orthologous sequences are also present in *Oryza sativa* indica 93-11, and wild relatives of rice, *Oryza glaberrima*, *Oryza punctata*, *Oryza minuta*, and *Oryza officinalis* (data not shown). However, no matching transcript could be found in the rice EST collections. The deduced protein sequence is 332 aa in length, and is 56.3% identical with P1-wr of maize. Compared to P1-wr, the first 150 aa containing the R2R3 Myb-domain are highly conserved, while the C-terminus is more variable. Synteny among flanking genes is well maintained. They are present in rice in the same order and orientation, but spread over a region of only 82 kb. As expected, fragmented or pseudo-genes present around the *P1-wr* cluster in maize are absent from the corresponding “*p*” region of rice.

The syntenic region in *Sorghum bicolor*, inbred line BTx623, is located on the long arm of chromosome 1 (about 74 Mb total length) between 61.203 Mb and 61.309 Mb [26]. The genomic arrangement in sorghum is more complex than in rice, as it contains three *p1*-homologous genes. However, the collinearity of flanking genes is well conserved. A functional *p1* ortholog, designated *y1* (*yellow seed 1*), was described in inbred line CS8110419 [27],[28]. The *y1* gene encodes a protein of 383 aa in length that is 68.5% identical at the amino acid level with P1-wr. In the inbred line BTx623 that was sequenced and used in our analysis *y1* is partially deleted [29]. The 3,218 bp deletion includes 5′ non-transcribed sequences, exon 1, intron 1, exon 2, and parts of intron 2. The *y1* gene in BTx623 is a null allele that is not transcribed [29]. A second *p1*-homologous pseudogene, *y2*, is located 3′ of *y1* in direct orientation [27]. The *y2* gene, which is nearly identical in lines BTx623 and CS8110419, shows multiple indels of various sizes compared to *y1*. The largest deletion includes intron 1, exon 2, and part of intron 2, whereas a smaller deletion removed the putative translation initiation codon. The *y2* transcripts were not detected by RT-PCR [27].

The investigated region contains a third gene copy that is homologous to the maize *p1* gene. This putative gene, which we named *y3* (*yellow seed 3*) in accordance with *y1* and *y2*, is located 29,877 bp upstream of *y1*. Based on RT-PCR experiments, *y3* is expressed in panicles at the time of anthesis (data not shown). Due to the lack of full-length transcript data, we deduced the exon-intron structure of *y3* from sequence alignments with *y1*. The largest ORF of *y3* potentially encodes a protein of 356 aa in length that is 58% and 61.2% identical at the amino acid level to Y1 and P1-wr-1, respectively. While the R2/R3 Myb domain and the acidic activation domain of Y3 are intact, a duplication of 146 bp in exon 3 and a truncation of 192 bp compared to *y1* at the 3′ end could render *y3* also nonfunctional because based on the white seed phenotype of BTx623 *y3* cannot substitute for *y1*. Surprisingly, the sequence 280 bp upstream the transcription start site that potentially carries promoter elements is well conserved in blocks compared to *y1*, *p2* from parviglumis and maize, with an overall nucleotide identity of 70%. The 3′ non-transcribed region has no similarity to *y1*, *y2*, *p2* and *P1-wr*, which could be a continuation of the deletion of the 3′ coding sequence.

The sequence separating *y1* and *y3* mostly consists of repetitive elements, but also harbors a gene 4,552 bp 3′ of *y3* that encodes a conserved hypothetical protein of 178 aa in size. This gene is duplicated 23,459 bp upstream of *y3* showing 91.2% nucleotide identity in a sequence that corresponds to the coding sequence of the downstream copy. However, this duplicated sequence is most likely a pseudogene, because indels cause several reading frame shifts resulting in a premature stop codon. Because both genes of unknown function and two *y* genes are arranged in an alternating pattern, they were probably duplicated together. To establish the order of duplication events that led from an ancestral *y* gene to three *y* copies we constructed a phylogenetic tree based on the coding sequences of exon 3. We were limited to exon 3 in our phylogenetic analysis, because exon 1 is partially deleted and exon 2 is completely deleted in *y2*. In addition to sorghum *y* genes, we included the *p* orthologous sequence from rice, *p1*, *p2* and the homoeologous *p* gene from maize (see below) in our multiple sequence alignments. The phylogenetic tree (not shown) reveals that the duplication of the ancestral *y* gene generated *y3* and the *y* copy that in a second duplication event gave rise to *y1* and *y2*. Possible recombination sites flanking the *y* genes could not be identified. Presumably, the intergenic spaces were completely reshuffled by transposable elements since the duplications occurred, leading to a loss or change beyond recognition of recombination sites. The phylogenetic analysis also reveals that the sorghum *y1*, *y2* and *y3* genes, and the maize *p1* (namely *P1-wr-1 and P1-rr* alleles) and *p2* genes cluster together. This is consistent with the fact that paralogous sequences arose after maize and sorghum shared their last common ancestor. Therefore, the amplification of *p* genes in maize and *y* genes in sorghum represent independent, parallel events. This finding is supported by the computed duplication times of *p1* and *p2* (2.75 mya [17]) and *y1* and *y2* (9.08 to 11.3 mya [27]) that are both younger than the maize and sorghum divergence time (11.9 mya) [30].

As maize originated from an allotetraploid event [18], we wanted to see whether a *p*-like sequence is retained on a homoeologous chromosome segment. Indeed, we found a highly similar sequence on the long arm of chromosome 9, which we named *p3 (pericarp 3)* consistent with the homoeologous copies *p1* and *p2*. According to EST data (GenBank accessions EB702996, EB702997) derived from mixed tissue (silks, husks, ears, pollen, shoot tips, leaf, root tips, whole seed, embryo), *p3* is transcribed. We verified the expression of *p3* in silk tissue using RT-PCR (data not shown). The coding sequences of *P1-wr* and *p3* share 87.3% nucleotide sequence identity. The deduced protein is 344 aa long and has an overall amino acid identity with P1-wr of 79.9%. The sequence from the transcription start site until position -312 bp that potentially contains promoter elements such as the TATA box is well conserved in *p3* compared to *y3*, *y1* and *p2*. Genes bordering *p3* on the centromere side are collinear with rice, sorghum and maize chr1. However, there is a break in synteny at the telomere end. Six genes are missing in this chromosome segment before synteny with rice, sorghum and maize chr1 resumes again (data not shown). While rice appears to have a relatively stable *p* locus, sorghum exhibits some degree of genome instability at its *y* locus. Clearly, in respect to ancient and recent genome instability the duplicated maize *p* loci stand out. Moreover, among all the maize *p1* alleles, the *P1-wr* allele underwent the most complex series of chromosomal changes.

## Discussion

### The *p2/p1* gene formed by illegitimate recombination

The filler DNA in the second intron of *p2/p1* is evidence for a DNA DSB and its repair. Presumably the DSB was repaired via the NHEJ pathway that resulted in the deletion of the 3′ end of *p2* (from part of the second intron and the complete third exon) and the 5′ end of a *P1-wr* unit (until part of the second intron), incorporation of filler DNA and ligation of the broken ends (Figure 6). The current configuration can be used to determine the size, origin and complexity of the inserted filler DNA. The deletion size may depend on the kind of break-inducing mutagenic agent, on exonuclease activity, and efficiency of DNA end protection. Previously reported NHEJ deletion sizes in maize genes range from 340 bp in *bz1* [31], 60 to 980 bp for *wx* [32], and 400 to 4,300 bp in *R-r* deletion derivatives [33]. Although a deletion size of about 10 kb for *p2/p1* would be the largest documented so far, the size seems to be in accordance with the examples above. Filler DNA is derived from sequences close to the deletion endpoint. It can be simple or complex, meaning a mosaic of different sequences of different origin. The filler DNA in *p2/p1* is 50-bp in size and is copied from two sites. Filler DNA can be as small as 8 bp as observed in *bz1* [31], or 1–131 bp as in *wx* [32], or 31 to 84 bp in *R-r* [33]. Only the filler DNA in *R-r* is of complex origin.

DSB repair via NHEJ mostly results in a recombination event that links two previously separated sequences. Therefore, the mechanism of NHEJ can be a source for creating new genes, as demonstrated here. Dependent on the size of the repair-induced deletion and distance between neighboring genes, two adjacent genes with the same transcriptional orientation can be merged, thus forming a novel hybrid gene. Based on the joining site, the fusion event may add or replace regulatory sequences as well as add or delete exons and introns. Here, we describe the likely fusion of two paralogous sequences with high sequence similarity. The new hybrid gene *p2/p1* was produced from almost identical, recently duplicated, genes that exhibit the same exon-intron structure but vary in their regulatory sequences. The deletion end points in both genes happened to be in the second intron, but at different positions. Therefore, the hybrid gene *p2/p1* maintains the overall gene structure of the parental genes, but slightly differs from *p2* in the third exon. Due to the high sequence similarity between *p2* and *P1-wr* in exon 1 and 2, the deduced gene product of *p2/p1* is identical with P1-wr. However, because P2 and P1-wr are almost identical and interchangeable, a functional change at the *p* cluster did not occur or is minimal. Despite the fact that the described fusion event has most likely no impact on the *p* cluster and downstream genes, this is, to our knowledge, the first evidence that NHEJ is linked to the formation of new, and functional genes in plants.

While DSBs in meiosis are solely repaired by homologous recombination, NHEJ is the repair pathway for DSBs in somatic tissue. All premeiotic events will only have an impact on evolution, when induced changes are transmitted to the offspring. Therefore, the importance of NHEJ-associated gene rearrangements depends on the amount of repaired DSBs in somatic tissues and the fraction of those events that are passed through meiosis. Each observed filler DNA in a maize population represents a somatic clonal sector in meristematic tissue that gave rise to the plant germline. Although filler DNAs as visible markers of a DSB repair event have been reported in only few maize loci, we hypothesize that they are rather widespread in the maize genome. For instance, filler DNAs were identified in various other *p1* alleles (unpublished). Successful searches for genomic rearrangements and filler DNAs depend on the comparison of the sequence of interest with a reference allele/locus of known lineage. However, in absence of such a reference, detection of filler DNA requires intragenomic sequence alignments, probably leading to an underestimation of the importance of NHEJ for plant evolution.

### Transposable elements in the *P1-wr* cluster

Transposable elements have shaped plant genomes in various ways (reviewed in [2],[9],[10]). Most obviously, amplification of mobile elements, especially LTR retrotransposons, led to genome enlargement, to differentiation among species and homoeologous chromosomes, and even to allelic variation. This extensive expansion and contraction due to LTR retrotransposon insertions can also be observed at the *P1-wr* cluster. Insertions of nine LTR retrotransposons into the *p1/p2* gene, which occurred between 1.4 and 0.2 mya, resulted into a 68-kb expansion of the *P1-wr* cluster (Figure 7). Transposition in a mostly nested fashion fragmented four LTR retrotransposons while five remained intact. Among nine elements, *Opie* and *Diguus* are present twice. Retroelement insertions into the *p* locus resemble transposition events at the *r/b* loci of maize in terms of insertion times and structure [34]. Only one solo LTR has been detected within this cluster. Solo LTRs can be generated by ectopic and intra-element recombination. DSBs between two repeat sequences are frequently repaired by intrachromosomal/intra-element recombination due to homologous sequences in close proximity to the DNA break [4]. Intra-element recombination events result in the deletion of one LTR and sequences in between them. Therefore, the TSD flanking the solo LTR are identical. Unequal crossovers involving homologous LTRs from ectopic positions cause segmental duplication and deletion of sequences. Because the TSD are derived from two independent insertion events, the short flanking sequences of the remaining LTR are different. The *Diguus* solo LTR is the outcome of intra-element recombination, because the TSD delimiting the LTR are identical. In the past 1.4 million years, the expansion of the maize genome within the *p* cluster due to retroelement transposition prevails the contraction due to deletion by about ten fold (assuming that only internal *Diguus* sequences were removed). In general, the size increase of the *P1-wr* cluster reflects the expansion of the maize genome. The displacement of the 3′ UTR of *p1/p2* due to transposon insertions generates a distant site for homologous recombination separated by retroelements that may suppress recombination frequencies in surrounding regions.

### Do transposable elements influence *P1-wr* and *P1-rr* expression?

Transposon mutagenesis in sequences upstream of the coding region of *P1-rr* revealed promoter and enhancer regions important for *P1-rr* regulation [21],[35],[36] (Figure 4). The putative regulatory fragments have been further characterized with a GUS reporter gene and tested in transient expression assays and in stably transformed plants [21]. Most of the promoter region and the putative proximal enhancer in *P1-wr* as well as *P1-rr* are composed of two transposable elements, namely 971 bp of an unknown *Mu-like* element (Mule, see also Text S1) and 107 bp of a MITE, separated by a unique 122-bp sequence. The question arises which of the three fractions contains the actual *cis*-acting element? It is tempting to speculate that the Mule contributes to the enhancer function in the *p1* gene. TIRs of *Mutator* elements include regulatory sequences that are required for the transcription of its *mudrA* and *mudrB* genes. Although this Mule has not been functionally characterized, it is likely that the 425 bp TIRs harbor regulatory sites in addition to the ones used by the tRNA lys gene. The Mule captured a gene fragment exactly between the TIRs, which is derived from the first intron of a calcium-dependent protein kinase gene located on chromosome 10. It is also possible that this acquired sequence is the source of the enhancer function.

*P1-wr* was shown to be posttranscriptionally silenced [22] possibly due to a tissue-specific repeat-induced gene-silencing (TRIGS) mechanism [14]. Is a transposable element conceivably involved in such a TRIGS mechanism? Transposons are subject to epigenetic silencing and most often dormant. TIRs of inactive *Mu* elements are heavily methylated which prevents transcription from the embedded promoter sites. Assuming that the Mule carries the proximal enhancer sequence, would silencing also contribute to *P1-wr* suppression? Is it possible that the same transposon sequence has activating and depressing functions dependent on the *p1* allele? While *P1-rr* may benefit from the transposon sequence by an increase in expression, epigenetically silenced alleles such as *P1-wr* and *P1-pr* [22],[37] may fall victim to transposon silencing. Interestingly, compared to *P1-rr*, the promoter and upstream regions of *P1-wr* are extensively methylated at *Hpa*II/*Msp*I sites [14].

### Are *p* homologs amplified in rice and sorghum?

**Figure 8:**
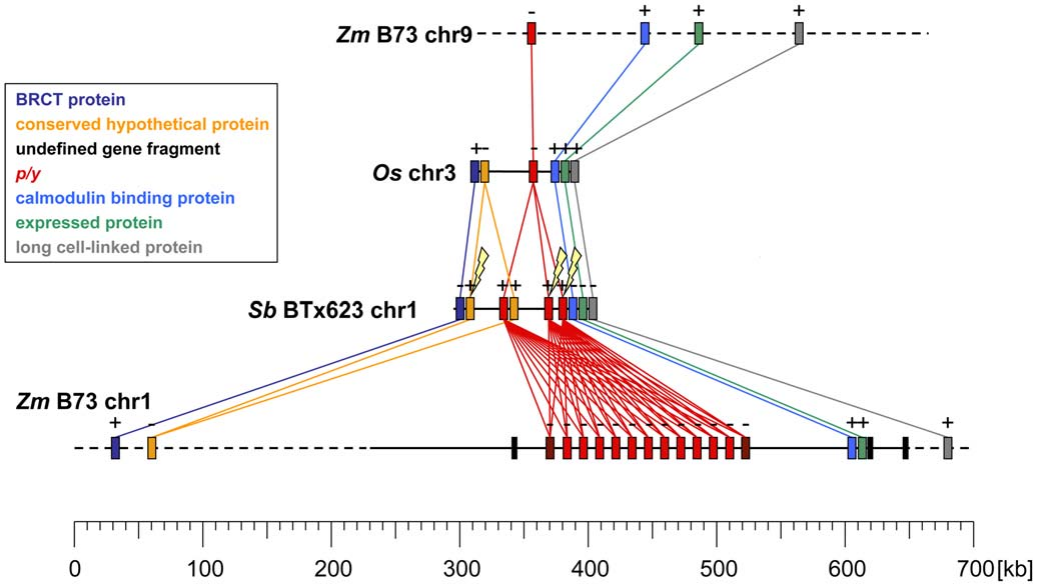
Synteny is maintained in maize, sorghum, and rice. The genomic comparison is centered around the maize *p* genes and its orthologs and includes five flanking genes. Chromosome segments of maize, rice and sorghum that contain *p* or orthologous genes are mostly collinear. However, a break in synteny occurred adjacent to the *p* orthologous gene on maize chr. 9. While rice has only one *p* ortholog, *y* and *p* genes in sorghum and maize chr. 1 are amplified. A gene encoding a conserved hypothetical protein in sorghum is duplicated as well. The expansion of the maize genome compared to rice and sorghum is most obvious even in this small genomic region. Genes are displayed as color-coded rectangles, and their transcriptional orientation is indicated by + and −. Undefined gene fragments are depicted in black. A dotted line indicates a sequence that consists of several contigs. Therefore, orientation of and distance between genes located in these contigs cannot be determined as of now. The gene orientation chosen in this figure is based on orthologous rice and sorghum genes. Lightning bolts stand for mutation events that generated pseudogenes.

We located *p* orthologous sequences in the rice and sorghum genomes, and confirmed their homology by descent with their syntenic positions. The rice genome contains only a single *p* orthologous sequence on the short arm of chromosome three. In contrast to the single *p* gene in rice, the sorghum-inbred line BTx623 contains three *p* equivalents (*y* genes) in a tandem array with *y1* and *y2* as non-functional deletion derivatives. Y3 does not rescue the colorless mutant phenotype of BTx623, indicating that Y3 is possibly non-functional as well. Alternatively, *y3* could be expressed in different tissues similar to *p1* and *p2*. Or Y3 has simply a totally dissimilar function. Two features of the grass genomes become immediately apparent (Figure 8). Although rice and maize diverged about 50 million years ago, the gene order between rice, sorghum and maize chromosome 1 and 9 is well conserved at the *p* orthologous regions. The only break in synteny occurred at maize chromosome 9 due to the removal of six genes (two missing genes in Figure 8) flanking the *p* homoeolog. The second characteristic feature is the overall genome expansion from rice to maize. The presence of two *p*-like genes in a syntenic arrangement of neighboring genes on different maize chromosomes is the result of allotetraploidization [18]. The intergenic space in maize is increased compared to sorghum and rice due to LTR retrotransposon insertions [38]. Tandem duplications as seen here at the *P1-wr* cluster in maize also contribute to the growth of the genome size.

### Amplification of *p*-like genes in maize

The amplification of *p*-homologous genes in maize occurred in three steps at different times. Because of allotetraploidization, the first duplication of *p* progenitors took place with the hybridization of both parental genomes. As shown here, both ancestral *p*-like genes are retained in the modern maize genome. While *p* is well characterized, the function of its homoeologous counterpart on chromosome 9 is not known. While P1 is the only transcription factor required in the phlobaphene biosynthesis pathway [39], it together with P2 is also involved in maysin accumulation. Maysin is a C-glycosyl flavone found in silk that confers resistance to corn earworm (*Helicoverpa zea*, Boddie) [40]. So far, no QTL for maysin synthesis has been reported at the chromosomal position of *p3* [40],[41]. The second *p* amplification happened by an unknown event as the result of a single gene duplication in contrast to whole genome duplication (WGD) [17]. It could have been the result of recombination between small homologous sequences such as MITEs, frequently found in or near genes [26]. This recombination event almost duplicated the entire gene because a site in an intergenic region approximately 5 kb downstream of the last exon was linked to a sequence 100 bp upstream of the transcription start site. The recombination sites can be inferred by aligning current sequences in the *P1-wr* cluster, because flanking sequences are usually maintained in unequal crossover, whereas gene numbers can change. Alternatively, one can envision that the truncated Mule caused a DNA DSB upon excision and initiated a repair/recombination event resulting in the duplication of the ancestral *p* gene. The Mule, which is absent in the proposed structure of an ancestral *p* gene, can be found at the putative recombination junction supporting this possibility. For example, an aberrant transposition event could have destabilized the *p1* and *p2* progenitor. Typically, a defective copy of the transposable element that triggered the genomic instability stays at the restructured site [33]. Although a mechanism for such gene duplication is not known, this model for initial amplification is more convincing than unequal crossover, especially because homologous sequences at the recombination junctions have not been detected. Not only would have the transposable element initiated a gene copying event but also added enhancer functions for *p1* alleles. Insertion of this transposon 100 bp upstream of the transcription start site could have activated and/or altered the expression pattern of the second copy now known as *p1*. A DNA fragment within a 400-bp sequence 3.8 kb further upstream in the intergenic region was recruited as a distal enhancer element for *p1*. This scenario would provide a model how transposable elements directly would contribute to the subfunctionalization of gene copies.

Similar to the first amplification step by polyploidization, none of the duplicated genes were removed from the genome or turned non-functional. *p1* and *p2* are the principle regulatory genes of the flavone pathway necessary for maysin synthesis. Although under distinct tissue-specific regulation *p1* and *p2* encode exchangeable products [42]. Based on our model, which is enhanced by the *p2-t* sequence from teosinte, the progenitor of modern maize, the ancestral *p* gene might have had a similar expression pattern as *p2*. Although *p2* is expressed in silk and anthers, *p2* does not confer phlobaphene pigmentation to any floral tissue, including pericarp and cob glumes. Therefore, it is feasible that the ancestral *p* gene primarily evolved in maize to protect silk and anthers from corn earworm damage. With *p1* acquiring new regulatory sequences due to the tandem duplication, expression of *p1* was extended to additional tissues such as pericarp and cob glumes where it is involved in pigmentation as secondary function. Accordingly, the tandem duplication appears to have resulted in subfunctionalization of the original function of the ancestral *p* gene.

The third amplification of *p* genes is allele-specific. While single copy *p1* alleles were characterized before [12],[15], multicopy alleles such as *P1-wr* are less well studied. In the B73 inbred line, amplification in tandem increased the *P1-wr* copy number to eleven, excluding the *p1/p2* hybrid gene. Due to their high sequence identity, tandem arrays could misalign in meiosis, which adds or deletes copies in case of a crossover event. Again, recombination is initiated by DNA DSBs, and its mechanism can be explained with the double-strand break repair (DSBR) model [43],[44]. The tandem repeats are not only subject to reciprocal unequal crossover, but also unidirectional gene conversion, where the acceptor sequence is replaced by a sequence copied from a donor. Gene conversion could homogenize paralogous gene copies, consistent with concerted evolution. While none of the *P1-wr* repeats are identical, stretches of several kb in size share polymorphisms between two or more copies (Figure 5). The length of potential conversion tracts at the *P1-wr* repeats is supported by data derived from the *bz* locus, where conversion tracts are between 1 and 1.5 kb long [45]. Tandem duplications generate diversity due to the process of unequal recombination. Because tandem arrays are common in plant genomes, their effects on evolution are quite significant. Tandem arrays giving rise to novel alleles were reported for numerous maize loci such as the 27-kDa zein [46], *kn1* [47], *pl1* [48], *a1* [49], *R-r* [33], *R-st* [50], *rp1* [51] and *rp3* [52].

### Model for *P1-wr* evolution

Any model for the evolution from a simple ancestral *p* gene to the complex multigenic *P1-wr* cluster has to explain (1) the creation of *p1* and *p2*, including their distinct expression pattern, (2) the formation of the *p2/p1* hybrid gene, (3) the amplification of *P1-wr* copies, (4) the formation of the *p1/p2* hybrid gene, and (5) the existence of a *p*-homologous sequence separated from the main complex. At least several DNA breaks were required to remodel the *p* locus. Mechanisms that involve DNA DSBs either as cause or consequence are homologous recombination (crossovers as well as gene conversion), illegitimate recombination, and transposition. Here, we can expand on a previously published model [17] to elucidate the evolution of the *P1-wr* allele.

The duplication of an ancestral *p* gene gave rise to the *p2* and *p1* genes that encode functionally interchangeable proteins [42]. However, both genes differ in their regulatory sequences and hence in their tissue-specific expression. Two *p* copies in direct orientation were subject to unequal crossover, creating a third gene. Additional unequal crossover events amplified the *P1-wr* genes until the cluster reached today's copy number. In addition, gene conversion took place resulting in homogenized *P1-wr* copies. Similarly, the *p1/p2* hybrid gene was generated by unequal crossover or by gene conversion.

A retroelement transposition into the 3′ UTR of the distal *p1/p2* gene, followed by additional nested insertions of multiple LTR retrotransposons, pushed the *p1/p2* end 68 kb apart (Figure 7). At the 5′ end of the cluster, a DNA DSB, potentially triggered by a transposon excision, occurred, which resulted in the deletion of the final 2 kb of *p2* (including the third exon) and 8 kb of the neighboring *P1-wr* repeat (or 8 kb plus increments of *P1-wr*-repeats). The DSB was repaired by NHEJ as evidenced by filler-DNA at the junction sequence (Figure 6).

The chronological order of all events in the proposed model can only partially be reconstructed. The two maize progenitors hybridized 4.8 mya [30], leading to *p2* on chromosome 1 and *p3* on chromosome 9. The first paralogous copy on chromosome 1 was produced 2.75 mya [17] and the earliest transposition 1.4 mya in *p1/p2*. Obviously, the *p1/p2* hybrid gene or at least the *p2* end had to be generated between these time points. The *p1* and *p2* genes had sufficient time to diverge and later on amplify. However, we cannot make any extrapolation on when the modification at the 5′ end occurred.

## Methods

### Plant material

Our *Ufo1* stock (X03G) and the inbred lines B73 and 4Co63 were obtained from the Maize Genetics Cooperation Stock Center (http://maizecoop.cropsci.uiuc.edu/) collection. *Ufo1* plants carry an undefined *P1-wr* allele. Therefore, *Ufo1* plants were crossed to plants from the 4Co63 inbred line that contain the *p1-ww* null allele. F1 plants were selfed and F2 plants were selected that are homozygous for *p1-ww* and display a stunted *Ufo1* plant phenotype. These plants were crossed to B73, and the resulting F1 plants carrying the B73 *P1-wr* allele and the 4Co63 *p1-ww* allele were used for our transcript analysis.

### Identification of BAC clones

The inbred line B73 contains a *P1-wr* allele, according to the colorless pericarp and red cob phenotype of B73 ears. Southern blot hybridizations, using the *p1*-specific probe 15 [24], reveal that the *P1-wr* allele from B73 is composed of a similar repeat structure as *P1-wr* from the previously studied inbred line W23 (data not shown) [14],[16]. Two publicly available BAC libraries constructed from B73 [53] were screened by hybridizing filters with probes 15 and 8B [24]. Whereas probe 15 is derived from a distal enhancer fragment of *P1-rr*, which is unique to *p1* alleles, probe 8B is obtained from the second intron of *P1-rr* and detects both *p1* and *p2*. To minimize the *P1-wr* repeats per BAC, the strategy has been to pick minimally overlapping BACs that include only a subset of *P1-wr* repeats.

Twenty-one BACs from two genomic libraries were isolated and further characterized. The presence of *p2* was evaluated by PCR using the primer pair p2-1 (p2-1F: ttacgcggcggcaggaaaatcacc, p2-1R: gacgcccaggccgcaggacag), which amplifies a 500-bp fragment about 650 bp upstream of the putative transcription start site of *p2-t*. In addition, insert size and both end sequences of each clone were determined and analyzed. BAC end sequencing was performed with the ABI PRISM BigDye Terminator Cycle Sequencing Ready Reaction kit and an ABI 3730 capillary sequencer (Applied BioSystems). BACs c0347H24, b0232A12, b0033H05 and b0086K13 were chosen for sequencing based on the above-mentioned criteria (Figure 2). Notice that the maize FPC maps (www.genome.arizona.edu/fpc/maize) were not available at the time of the library screenings. Currently, BAC c0347H24 is included in the HICF FPC map, and BACs b0232A12 and b0086K13 are integrated in the agarose FPC map (data not shown).

### Shotgun library construction and sequencing

BAC DNA was isolated using the Large-Construct Kit (QIAGEN). For shotgun library construction, the purified BAC DNA was physically sheared and then ligated into a pUC vector as previously described by [54]. Plasmid inserts were sequenced from both ends using universal primers [55], ABI 3730 capillary sequencers, and the ABI PRISM BigDye Terminator Cycle Sequencing Ready Reaction kit (Applied BioSystems). Base calling and assembly were carried out using phred/phrap programs [56]. About 10× sequence coverage was generated for each BAC. Sequence gaps were closed by primer walking or by transposon minilibraries (Finnzymes), constructed according to the manufacturer's instructions.

### *P1-wr* repeat subcloning, sequencing, and assembly/analysis

To assemble the *P1-wr* containing part of the BACs, the sequence of the entire plasmid insert from which the shotgun sequence originates was determined. However, the insert sequences could not unambiguously be assembled into just one large contig, because the amount of polymorphisms among the repeats is rather small and the average length of a plasmid insert is less than 4 kb, which is significantly shorter than the size of one *P1-wr* repeat. Therefore, each complete *P1-wr* repeat from BACs c0347H24, b0033H05 and b0086K13 was subcloned into the plasmid vector pBluescript II SK(+) (Stratagene) using the restriction endonucleases *Eco*RV or *Xho*I. Both enzymes release a full copy of a *P1-wr* repeat as they cut only once within a repeat. The unique sites were detected by assembling short (up to 800 bp) shotgun sequences derived from all *P1-wr* repeats. No polymorphisms were detected at their recognition sites. The individual clones were completely sequenced using primers that are spanning the entire repeat length (approximately one primer every 300 bp, primer sequences available upon request). The sequencing reactions were carried out with the ABI PRISM BigDye Terminator Cycle Sequencing Ready Reaction kit and analyzed on an ABI 3730 capillary sequencer (Applied BioSystems). The sequences were assembled and evaluated with the Lasergene software (DNAstar). The recognition sites of the endonucleases are about 5 kb apart. The repeat order was established based on polymorphisms within the overlapping fragments. The GenBank accession number for the entire 379-kb sequence is FJ614806.

### Genomic PCR and Reverse-Transcription PCR (RT–PCR)

Total RNA was extracted from pericarp tissue 20 days after pollination with the RNeasy Plant Mini Kit (Qiagen). RNA was reverse-transcribed to cDNA using the SuperScript First-Strand Synthesis System (Invitrogen) with oligo(dT) or random hexamer primers. cDNA was then PCR-amplified with four primer pairs that flank polymorphic sequences of all *P1-wr* repeats, *p2/p1* and *p1/p2*: p-hAT-ex2F GCGGGCGGGCTTGGACAGGAAACT, p-hAT-ex2R GGGTGGCGTGGAGCTTGATGATGA, p-ex1-3-1F TAACCGTGCGCAAGTAGTAGTG, p-ex1-3-1R GGCCCGGCGGTGTATTTC, p-ex3-1F CCACCTCCCCGGCCGAACAGACAA, p-ex3-1R GCTCCGGCCCGCCCCACAGATG, p-ex3-3F GGGGGAGGCCGACAGCGAGATG, p-ex3-3R ACCGGCGGGAGAACTACCTTTACA. As a control for presence and ratio of individual *P1-wr* repeats, genomic DNA corresponding to the RNA sample was PCR-amplified in parallel. 96 PCR and RT-PCR products per primer pair were cloned and also sequenced with universal primers. DNA sequences were analyzed with Lasergene (DNAstar).

### Sequence annotation

The maize sequence was manually annotated using homology searches in various GenBank databases with multiple BLAST programs (BLASTN, BLASTP, BLASTX, TBLASTX) [57]. By the same approach, existing annotations of the rice [58] and sorghum [26] sequences were manually adjusted where necessary.

### Phylogenetic analyses

All sequences were aligned using CLUSTALW (as implemented in MEGA4.0 [59]), and the alignment was manually adjusted. Phylogenetic analyses were conducted in MEGA4.0 [59]. The phylogenetic tree was inferred by using the Minimum Evolution (ME) method. The percentage of replicate trees in which the associated taxa clustered together in the bootstrap test (1000 replicates) was calculated. The evolutionary distances were computed using the Maximum Composite Likelihood method and are in the units of the number of base substitutions per site. The ME tree was searched using the Close-Neighbor-Interchange algorithm at the default search level. The Neighbor-joining algorithm was used to generate the initial tree. All positions containing gaps and missing data were eliminated from the dataset (Complete deletion option). There were a total of 449 positions in the final dataset. Similar trees were obtained by using the same alignments with the Neighbor-Joining (NJ) and Maximum Parsimony (MP) method.

The TSD sequence was used to identify a pair of LTRs that belong to the same LTR-retrotransposon. LTRs were aligned using ClustalX [60], and the resulting alignment was manually adjusted. Distance estimations between pairs of LTRs were based on Kimura's two-parameter model (K2P) as implemented in MEGA4.0 [59]. Using the formula T = K/2R, where T is time, K is the number of substitutions and R is the rate of substitutions per site per year, we calculated the time of LTR retrotransposon insertions within the *P1-wr* cluster (Figure 7). K was computed using the software MEGA4.0 [59]. We applied the substitution rate R of 1.3×10^−8^ mutations per site per year that is based on the average level of nucleotide substitutions in intergenic regions [61].

## Supporting Information

Figure S1 A *Mu*-like transposon is present in *p1* alleles but absent in *p2*. The alignment of *p1* and *p2* promoter sequences reveals the probable 5′ recombination site of the ancestral *p* gene duplication event. Interestingly, this site coincides with a *Mu*-like-transposon insertion present in *p1* but absent in *p2*. The alignment shows sequences upstream of the transcription start site containing the 3′ end of the transposon and promoter sequences as defined for *P1-rr*. The Mule transposon is shaded in yellow, its potential target site duplication (TSD) in blue. Sequences shaded in red differ from the consensus sequence. Notice the increase in polymorphisms in the transposon sequences. One deletion event includes TSD sequences. Only *P1-wr* repeats are included in this alignment that are polymorphic for this sequence.
(5.77 MB TIF)

Text S1 Supplemental material.
(0.07 MB DOC)
