# Supplementary figures and images for: Change of Gene Structure and Function by Non-Homologous End-Joining, Homologous Recombination, and Transposition of DNA
Source: PLoS Genet. 2009 Jun 12;5(6):e1000516. doi: 10.1371/journal.pgen.1000516 (PMC2686159; doi:10.1371/journal.pgen.1000516)

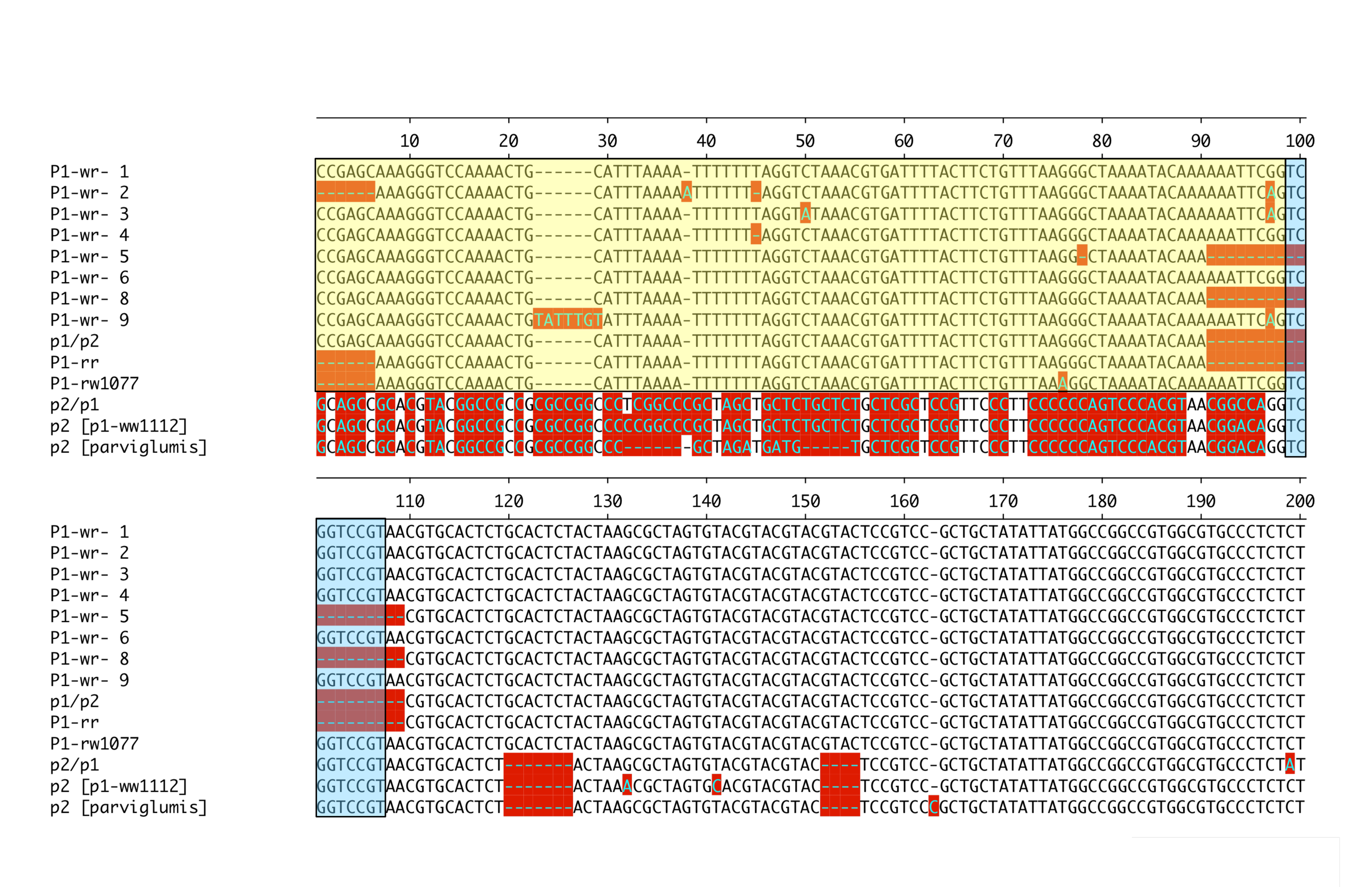

Supplement: Figure S1 — A Mu-like transposon is present in p1 alleles but absent in p2. The alignment of p1 and p2 promoter sequences reveals the probable 5′ recombination site of the ancestral p gene duplication event. Interestingly, this site coincides with a Mu-like-transposon insertion present in p1 but absent in p2. The alignment shows sequences upstream of the transcription start site containing the 3′ end of the transposon and promoter sequences as defined for P1-rr. The Mule transposon is shaded in yellow, its potential target site duplication (TSD) in blue. Sequences shaded in red differ from the consensus sequence. Notice the increase in polymorphisms in the transposon sequences. One deletion event includes TSD sequences. Only P1-wr repeats are included in this alignment that are polymorphic for this sequence. (5.77 MB TIF) [file pgen.1000516.s001.tif]
