# Supplementary material for: Change of Gene Structure and Function by Non-Homologous End-Joining, Homologous Recombination, and Transposition of DNA
Source: PLoS Genet. 2009 Jun 12;5(6):e1000516. doi: 10.1371/journal.pgen.1000516 (PMC2686159; doi:10.1371/journal.pgen.1000516)
Supplement: Text S1 — Supplemental material. (0.07 MB DOC) [file pgen.1000516.s002.doc]

**Text S1**

**Characterization of a *Mutator*-like sequence**

The *Mutator*-like transposon is flanked by two terminal inverted repeats (TIR) of about 425 bp starting with the sequence CCGAATTTTT. Each TIR contains seven subterminal inverted repeats (SIR) of 14 bp (GCTCGGCGCCATAG) that form the above-mentioned palindrome. Interestingly, each TIR carries a tRNA^lys^ located about 280 bp from both transposon termini. Each tRNA^Lys^ gene can be transcribed but does not encode a functional product. Upon insertion, the element generates a 9-bp target site duplication (TSD), which is characteristic for *Mu*-like elements. Based on EST evidence, two transcripts stem from the presumptive autonomous element. Measured relative to the 5’ end, the first transcript initiates at around position 800 and terminates at about 3950 bp. The transcript encodes a deduced protein of 864 aa that is similar to MURA, the transposase of *MuDR*. The second primary transcript is about 2,600 nt in length spanning from position 4,030 to 6,630. It encodes a protein of unknown function. Interestingly, a *Mu*-like transposable element in rice seems to encode only one protein that has an N-terminus similar to the first and a C-terminus similar to the second putative protein. However, this annotation in rice lacks EST support. If true, it prompts the question whether in rice a fusion protein was created with both coding sequences or whether in maize a coding sequence was split to generate two individual proteins with separate functions. Ultimately, genetic studies will be necessary to reveal the functions of the element-encoded proteins and their requirements for transposition of an autonomous element. Using BLASTN searches against maize BACs with different sequences from the transposon as queries, the copy number of this family is estimated to be several hundreds (approximately 500). While the majority of family members are deletion derivatives, about 100 elements potentially encode at least one putative protein. Similar to Pack-MULEs in rice [1], many if not most elements including deletion derivatives carry fragments captured from cellular genes.

We identified seven additional elements that carry the same insert at the same position as the element in the proximal enhancer region. It is not likely that eight independent sequence-capturing events resulted in the incorporation of the exact gene fragment at the same site. Instead, these elements amplified by transposition, which is also confirmed by the presence of TSD at the insertion sites. An increase in copy number can occur during the S phase of the cell cycle, when an element excises from a replicated site and inserts into a not yet replicated target sequence. Because all eight elements lack a transposase-encoding sequence, transposition had to take place in presence of an autonomous element that provided the transposase function. Most of the elements have accumulated insertions or deletions in the TIRs and are therefore most likely transposition-defective. Two out of eight elements also have a truncated insert. Deletions often result from aborted transposition events and aborted gap repairs.

The effect of this Mule on alternative splicing has been shown previously. A nearly complete element belonging to the same *Mu*-like family has been detected as an insert in the *ZmHox1a* gene [2]. This gene has been molecularly characterized, and although the authors inferred the presence of a transposable element based on sequence similarity to a *Mu* transposase, they did not clearly identify the transposon.

Although Mule elements acquiring gene fragments are frequently found in many plant species (see pack mules in rice) [1], the mechanism of gene capture remains unknown. Interestingly, the gene fragment is exactly inserted between both TIR, thereby replacing the internal transposon sequences. It will be interesting to see whether autonomous elements as well as derivatives are able to incorporate host sequences. Because the captured host sequence is derived from an intron sequence, any potential mechanism obviously does not rely on an RNA intermediate.

## *P1-wr* intron sequences

The exon sequences were found to be highly similar between *P1-wr* and *P1-rr* (Figure 4). Surprisingly, the intron sequences are very conserved as well. *P1-wr-1* is identical to *P1-rr* in the first intron, which is 120 bp in length. *P1-wr-1* and *P1-rr* are 98.7% identical in the second intron, which is 4,584 bp long. *P1-wr-1* and *P1-rr* vary in only three SNPs and seven indels, ranging from 2 bp to 27 bp. The composition of transposable elements and their remnants is indistinguishable between *P1-wr* and *P1-rr*. The MITE *Heartbreaker*, the repeat element Pilgrim, and a previously unidentified MITE are present in all *p* sequences. However, an unknown putative *hAT*-like element is unique to *P1-wr* and *P1-rr* (Figure 4). This transposon terminates in 12-bp imperfect TIR (CAG t/g GGCGG g/a a/t C) and generates an 8-bp Target Site Duplication (TSD) ATTTTGAC upon insertion. Interestingly, the 3’TIR is identical to a TIR from a rice *hAT*-like element and has only 1-bp mismatch compared to the TIR from *dTph1* of petunia. A *Stowaway* MITE, located at the 3’ end of intron 2, is present in *P1-wr* and *P1-rr*, but is missing in *p2* and *p2*-derivatives like *P1-rw*.

At least 1,365 bp or 29.8% in intron 2 from *P1-wr-1* are comprised of transposable elements (Figure 4). Aligning sequences derived from different alleles greatly facilitates the identification of already described and novel transposons. The characterization and classification of transposable elements is based on sequence identities and comparison of terminal inverted repeats in addition to length of target site duplications, but older ones may have deteriorated beyond recognition.

## The *p1/p2* chimeric gene

Due to the high sequence identity to *P1-wr* and *P1-rr*, *p1/p2* carries the same features including regulatory regions as described above for the homologous *P1-wr* repeats. It contains only one synonymous substitution in the first exon compared to *P1-wr* (99.8% nucleotide identity). *p1/p2* shows otherwise multiple sequence polymorphisms to *p2-t* (78.2%) and *p2-m* (73.7%), especially indels in the 5’ UTR. Two synonymous nucleotide substitutions compared to *P1-wr* and *P1-rr* can be found in the second exon. The second SNP is common to *p2-m*, *p2-t*, and *p2/p1*. From this SNP on, 704 bp after the transcription start site, the remaining *p1/p2* sequence is highly similar to *p2-m* (99.7% encompassing 7,669 bp) until the end of the available sequence.

Comparison of the coding region of the last exon with *P1-wr* and *P1-rr* reveals eight synonymous and nine non-synonymous nucleotide substitutions. Furthermore, 6-bp and 3-bp indels that maintain the reading frame cause the lack of three amino acids in *p1/p2* and *p2-m* compared to *P1-wr* and *P1-rr*. A stretch of 9 bp in *p1/p2 and p2-m*, but absent in *P1-wr* and *P1-rr*, leads to three additional alanines at the C-terminus of the hypothetical protein. None of the amino acid changes occur in the Myb domain, which is still encoded at the 5’end of the third exon. With the exception of a missing valine residue, due to the aforementioned 3-bp indel in *p1/p2,* and *p2-m*, the acidic activation domain is conserved among known P proteins as well. More polymorphisms can be found in the 3’UTR comparing *p1/p2* to other *p* sequences.

**Characterization of the *Ins2* transposable element**

*Ins2* terminates in 14-bp imperfect inverted repeats (TA T/G AGATGGCCAAA), and is flanked by 8-bp direct repeats (ACGGCCAC). BLASTN searches revealed that a similar insertion sequence was previously identified upstream of the coding region of the *bz1-R* allele [3]. *Ins2* was also found in the promoter region of certain *y1* alleles [4,5] and in the 3’UTR of a *c1* allele [6]. This element is also present in transcripts as shown in BLAST searches of EST databases. *Ins2* is well conserved across species borders and *Ins2*-like sequences have been detected in rice. One randomly chosen member of rice (isolated from GenBank accession AC104845.2), for example, is 581 bp long and has an overall similarity to *Ins2* in *P1-wr* of 44.2 %. The rice *Ins2* is delineated by 21-bp perfect TIR (tatagatggccaaaaggcccg), which are identical in the initial 14 bp to the maize *Ins2*. Like in maize, the rice *Ins2* is bordered by 8-bp direct repeats and is highly abundant based on BLASTN searches.

**References**

1. Jiang N, Bao Z, Zhang X, Eddy SR, Wessler SR (2004) Pack-MULE transposable elements mediate gene evolution in plants. Nature 431: 569-573.

2. Comelli P, König J, Werr W (1999) Alternative splicing of two leading exons partitions promoter activity between the coding regions of the maize homeobox gene Zmhox1a and Trap (transposon-associated protein). Plant Mol Biol 41: 615-625.

3. Ralston EJ, English JJ, Dooner HK (1988) Sequence of three *bronze* alleles of maize and correlation with the genetic fine structure. Genetics 119: 185-197.

4. Buckner B, Miguel PS, Janick-Buckner D, Bennetzen JL (1996) The *y1* gene of maize codes for phytoene synthase. Genetics 143: 479-488.

5. Palaisa KA, Morgante M, Williams M, Rafalski A (2003) Contrasting effects of selection on sequence diversity and linkage disequilibrium at two phytoene synthase loci. Plant Cell 15: 1795-1806.

6. Paz-Ares J, Ghosal D, Wienand U, Peterson PA, Saedler H (1987) The regulatory *c1* locus of *Zea mays* encodes a protein with homology to myb proto-oncogene products and with structural similarities to transcriptional activators. Embo J 6: 3553-3558.
